# Repeatability and correlation of physiological traits: Do ectotherms have a “thermal type”?

**DOI:** 10.1002/ece3.2632

**Published:** 2016-12-22

**Authors:** Celine T. Goulet, Michael B. Thompson, David G. Chapple

**Affiliations:** ^1^ School of Biological SciencesMonash UniversityClaytonVic.Australia; ^2^ School of Biological SciencesUniversity of SydneySydneyNSWAustralia

**Keywords:** individual variation, locomotor performance, selected body temperature, syndrome, thermal physiology

## Abstract

Across a range of taxa, individuals within a species differ in suites of correlated traits. These trait complexes, known as syndromes, can have dramatic evolutionary consequences as they do not evolve independently but rather as a unit. Current research focuses primarily on syndromes relating to aspects of behavior and life history. What is less clear is whether physiological traits also form a syndrome. We measured 10 thermal traits in the delicate skink, *Lampropholis delicata*, to test this idea. Repeatability was calculated and their across‐context correlations evaluated. Our results were in alignment with our predictions in that individual thermal traits varied consistently and were structured into a physiological syndrome, which we are referring to as the thermal behavior syndrome (TBS). Within this syndrome, lizards exhibited a “thermal type” with each being ranked along a cold–hot continuum. Hot types had faster sprint speeds and higher preferred body temperatures, whereas the opposite was true for cold types. We conclude that physiological traits may evolve as a single unit driven by the need to maintain optimal temperatures that enable fitness‐related behaviors to be maximized.

## 1 Introduction

Individual variation is essential for evolution to occur. It has the potential to link phenotype with fitness, forming the substrate upon which natural selection acts (Careau & Garland, 2012). The degree to which selection drives adaptive change is determined, in large part, by the magnitude and stability of such phenotypic differences (Brodie & Russell, 1999). Selection tends to be strongest when individuals express consistent values of a trait over time or circumstance (Brodie & Russell, 1999). This in turn leads to predictable fitness consequences (Pruitt, Riechert, & Jones, 2008). However, phenotypic evolution may be constrained when correlations among traits arise to form a syndrome (Sih, Bell, Johnson, & Ziemba, 2004). When traits covary, due to a shared underlying mechanism such as genetic, environmental, or neurobiological effects, they are no longer free to evolve in complete independence, but instead, selection works upon them as a single unit (Sih, Bell, Johnson, & Ziemba, 2004). This limited lability manifests itself ecologically into carryover effects whereby an individual's trait expression in one context (e.g., feeding, mating, or predator avoidance) or situation (e.g., environmental gradients or ontogenetic stages) is linked with its expression across functionally different contexts and/or situations (Stapley, 2006). As a consequence, each individual's phenotypic response remains consistent despite changes in the current conditions.

Often, the covariation of traits producing a syndrome can have opposing effects on fitness, as the evolution of independent trait optima is prevented (Sih, Kats, & Maurer, 2003). Individuals are no longer free to respond optimally within all situations or contexts. Instead, each is restricted to the expression of a particular configuration of trait values, known as its trait type, and is positioned along a multidimensional axis representing the entire range of possible configurations. Under a given circumstance some trait types will gain fitness while others will decline (Sih, Bell, & Johnson, 2004). As an example, asocial individuals showing higher levels of aggression, activity, and tolerance toward conspecifics will gain greater resources and be better at avoiding predators than social types (Pruitt et al., 2008). However, they may also be inappropriately aggressive toward potential mates or engage in superfluous killing of prey which, in turn, reduces reproductive potential and energy stores. Selection for or against differing trait types may therefore vary according to ecological or environmental context. Accordingly, syndromes may play a key role in maintaining interindividual variation within a population while also serving to explain deviations from optimal trait expression (Stamps, 2007).

Several types of syndromes have recently been identified. They include the life‐history syndrome (Bergmans, 1984), dispersal syndrome (Clobert, Galliard, Cote, Meylan, & Massot, 2009), behavioral syndrome (Dingemanse & Wolf, 2010; Pruitt et al., 2008; Sih & del Giudice, 2012; Sih et al., 2003; Stamps & Groothuis, 2010), and invasion syndrome (Chapple, Simmonds, & Wong, 2012). Each of these describes correlations among various behavioral and/or life‐history traits such as aggression, boldness, activity, exploration, growth rate, reproductive strategy, and longevity. In stark contrast, only one, the Pace‐of‐Life Syndrome (Careau & Garland, 2012; le Galliard, Paquet, Cisel, Montes‐Poloni, & Franklin, 2012; Reale et al., 2010), incorporates physiological processes such as metabolism into its framework. It predicts that greater levels of activity, exploration, boldness, and aggressiveness occur in individuals that tend to be “fast” (high metabolic rate, fast maturation, low survival, and low investment to offspring quality) while the opposite suite of personality traits occurs in individuals that tend to be “slow” (Careau & Garland, 2012; le Galliard et al., 2012). This functional integration of exploratory behavior and resting metabolic rate is present in deer mice (*Peromyscus maniculatus*), where individuals that explore at high rates have correspondingly high energy expenditures compared to those that explore at low rates (Careau, Bininda‐Emonds, Thomas, Réale, & Humphries, 2009).

Given the strong and positive relationship between metabolism and temperature, it seems plausible that, analogous to the Pace‐of‐Life Syndrome, thermal behavior could potentially be structured into a syndrome as well (Clarke & Fraser, 2004; Pruitt, Demes, & Dittrich‐Reed, 2011). Under this premise, traits regarding thermoregulation and performance would be correlated to form distinct thermal types. Each thermal type would be ranked along a cold–hot continuum where some individuals (cold types) would select and perform best at low body temperatures (*T*_b_) while others (hot types) would select and perform best at high *T*_b_'s. This syndrome, termed here as the Thermal Behavior Syndrome (TBS), would be particularly relevant among ectothermic species given the strong influence temperature plays in nearly all of their biological processes (Angilletta, 2009). Countering the coadaptation theory which states that (1) performance would be maximized at temperatures that an individual experiences during normal activity; and (2) natural selection would favor individuals whose optimal performance temperature matches their preferred body temperature (Angilletta, Niewiarowski, & Navas, 2002), the TBS, instead, predicts that individuals would not exhibit a high level of plasticity, but rather maintain their thermal type across contexts (e.g., digestion, reproduction, or ecdysis) or situations (e.g., ontogeny or season), despite the theoretical need to undergo physiological adjustments which optimize their current state.

In support of the proposed TBS, empirical data demonstrate that ectothermic individuals do vary systematically with respect to their thermal physiology (Table S1). These studies reveal that intra‐individual variation among thermal traits is temporally consistent (repeatable) over both the short term and long term. In some traits, namely preferred and optimal performance temperatures, correlations do emerge (Artacho, Jouanneau, & le Galliard, 2013; Bauwens, Garland, Castilla, & Van Damme, 1995). Nonetheless, it has yet to be determined whether both within‐ and between‐individual correlations among physiological traits are maintained across different situations or contexts. Without such information it remains unclear if thermal traits are indeed configured into a syndrome.

Here we employed a syndrome approach to test our TBS hypothesis within a population of delicate skinks, *Lampropholis delicata* (De Vis 1888). Repeatability in sprint speed, locomotory performance, and thermal preference was quantified under two situations: initial entry into the laboratory and again after 9 weeks of laboratory acclimation. These two situations served to represent a distinct set of environmental conditions (unpredictable and fluctuating natural conditions vs. constant laboratory conditions) from which comparisons of physiological responses could be evaluated. Repeatability of sprint speed was also assessed across temperatures to further text for contextual stability. If the TBS were to exist, individuals should respond to their recent experience of climatic fluctuation by exhibiting lower within‐individual variation vs. among‐individual variation in their thermal behavioral traits whereby each lizard would maintain its respective thermal type across situations. Alternatively, if there is no TBS, thermal trait values of all individuals would instead converge as they all would become physiologically adapted to the standard laboratory temperature conditions (i.e., acclimatization).

The delicate skink is an excellent model to test the TBS concept because interindividual variation among behavioral traits (exploration: *r *=* *.33, *p *=* *.005; activity: *r *=* *.31, *p *=* *.009: and sociability: *r *=* *.32, *p *=* *.008) is quite conspicuous in this species (Chapple, Simmonds, & Wong, 2011; Michelangeli, Wong, & Chapple, 2016; Moule, Michelangeli, Thompson, & Chapple, 2016). As the first of its kind, this study could reveal mechanisms extending beyond behavior and life history as the drivers of interindividual variation and its associated ecological consequences.

## 2 Materials and Methods

### 2.1 Study species and field collection

**Figure 1:**
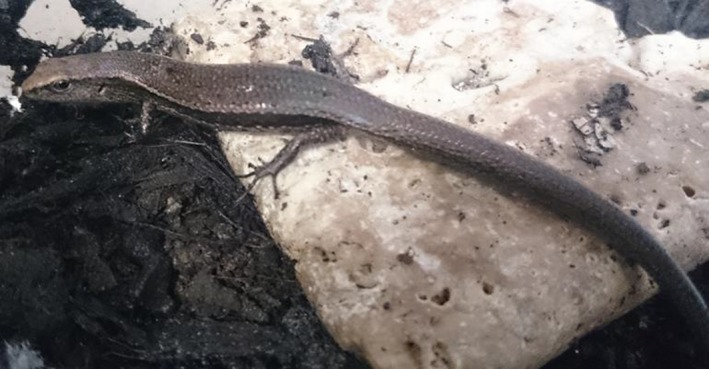
Photograph of a male delicate skink (*Lampropholis delicata*)

The delicate skink is a small [35–55 mm adult snout–vent length (SVL)], heliothermic, insectivore that is locally abundant and geographically widespread in eastern Australia (Chapple, Hoskin, Chapple, & Thompson, 2011b) (Figure 1). Its distributional range spans 26° of latitude from north Queensland to southern Tasmania (Chapple, Hoskin, Chapple, & Thompson, 2011a). Common habitat associations include moist habitats, including such as rainforests, wet sclerophyll forests, woodlands, and heaths. Thirty adult male lizards with complete tails were collected from the Brisbane region (Queensland, Australia: 27°38S 153°05E) between October and December 2013. Each was individually marked with a unique Visible Implant Elastomer (Northwest Marine Technology, Inc. Washington, USA) color code and transported back to the animal housing facility at Monash University (Clayton, Victoria, Australia). Lizards were held in groups of four and maintained at 20°C with a 14‐h light: 10‐h dark cycle (0600–2,000 hr). Basking lamps created a thermal gradient of 20 to 35°C to promote natural thermoregulatory behavior (Table 1). Lizards were fed crickets (*Acheta domesticus*) three times weekly and provided water ad libitum.

**Table 1:** Summary statistics and temporal repeatability (τ) of locomotory performance. Sprint speed values are in cm/second, *T*_set_ and *B*_80_ are in number of degrees (°C), and all other traits are in °C. Repeatability estimates in boldface are significant at *p *<* *.05

Variable	Mean		Standard Dev		Minimum		Maximum		τ	*p* Value
	Time 1	Time 2	Time 1	Time 2	Time 1	Time 2	Time 1	Time 2		
Mean velocity	29.35	29.33	4.65	5.02	21.44	19.60	38.86	41.05	**0.33**	<.001
*V*_max_	58.42	54.88	14.10	14.63	27.72	26.82	89.29	95.42	**0.04**	.32
LB_80_	26.39	25.44	3.58	3.31	20.82	21.34	32.71	32.16	0.00	.39
UB_80_	35.06	35.51	1.90	1.53	28.01	28.82	36.23	36.23	−0.01	.80
*B*_80_	8.67	10.07	3.56	3.33	3.45	3.91	14.53	14.45	0.00	.48
*T*_opt_	30.72	30.47	2.25	1.97	24.64	26.07	34.47	34.15	0.00	.47
*T*_sel_	25.84	26.42	2.05	1.72	17.16	21.00	27.96	28.14	**0.07**	.01
LT_set_	24.92	25.43	2.24	2.67	15.96	16.76	27.94	28.08	**0.05**	.03
UT_set_	26.87	27.59	2.43	1.45	17.50	24.65	31.40	29.88	0.04	.06
*T*_set_	1.96	2.16	1.53	2.00	0.00	0.19	6.61	8.12	−0.01	.69

### 2.2 Thermal behavior measurements

Lizards (*n *=* *28) were exposed to tests at two time intervals (Time 1 =* *initial entry into lab; Time 2 =* *9 weeks laboratory acclimation), quantifying aspects of locomotor performance and thermal preferences to determine the existence of a physiological syndrome. Seventy‐two hours separated test days to avoid interactions among experimental responses. All tests were performed when lizards were in a postabsorptive state (2 days without food) (van Berkum, Huey, Tsuji, & Garland, 1989). Interlimb length (ILL), SVL, and mass were measured prior to each test to assess body size effects. Four lizards died during the course of the experiment, and thus, their associated data were removed from all analyses.

### 2.3 Sprint speed and locomotory performance

Lizards were raced down a 1‐m long × 10‐cm wide racetrack (as per Cromie & Chapple, 2012) at five temperatures (15, 20, 25, 30, and 35°C) in a random order. Lizards were tested at a single temperature three times each test day with at least 30 min between successive runs. Prior to the first trial and in between trials, lizards were placed into a thermal chamber set to the race temperature for at least 15 min. Sprint speed was determined by infrared sensors positioned at 25‐cm intervals. Each race produced a velocity measurement for each of the four segments between the sensors. The fastest segment was considered an individual's maximal velocity (*V*_max_) and the average *V*_max_ at each temperature was its *V*_mean_. Maximum speed data at each temperature were then used to generate individual performance curves from which optimal performance temperature (*T*_opt_) defined as the *T*_b_ which maximizes performance, performance breadth (*B*_80_) defined as the range of *T*_b_'s over which lizards can perform ≥80% of their maximum speed, and the lower (LB_80_) and upper (UB_80_) bounds of the performance breadth were estimated. Critical thermal minima and maxima needed to construct the curves were based upon published data (Greer, 1989).

### 2.4 Thermal preferences

Lizards were placed into a 40 × 100 cm thigmo‐thermal gradient constructed of aluminum and partitioned into four equal runways. A near linear gradient ranging from 15 to 36°C was produced by hanging two 250‐W infrared bulbs at one end of the chamber and placing a cold plate beneath the other end. Because the delicate skink is heliothermic, infrared bulbs were used to eliminate the effect of light as a potential confounding factor. A row of iButton dataloggers (Alfa‐Tek Australia, Vic., Australia) spanned the length of each lane to measure surface temperature. Dataloggers were calibrated against a subset of lizards (*n *=* *10) to ensure they accurately reflected the *T*_b_'s of the lizards. For calibration, individual lizards were placed in the thermal gradient at each iButton position for 2 min. Body temperatures were recorded with a T‐type thermocouples taped (Leucopore ©) above their pelvic girdle (Camacho et al., 2014; Clusella‐Trullas, van Wyk, & Spotila, 2009). Two minutes was selected as Fraser and Grigg (1984) determined that the thermal time constant for this species was 1.33 min. Body temperatures were regressed onto iButton temperature to produce a regression equation from which *T*_b_ was calculated. At the onset of the test, lizards were placed individually into the cool end. After a 1‐h acclimation period, the locations of each lizard were monitored from 10:00 to 16:00 with video cameras positioned over the chamber. Given the short time constant of this species’ heating and cooling rates (1.30 ± 0.338 min; (Fraser & Grigg, 1984)), *T*_b_'s were inferred from the selected positions along the gradient. These data were used to calculate the following thermal preference measures for each individual: mean selected body temperature (Ernst, Creque, Orr, Hartsell, & Laemmerzahl, 2014), setpoint range (*T*_set_) defined as the central 50% of recorded *T*_b_'s, and lower (LT_set_) and upper (UT_set_) set‐point temperatures.

### 2.5 Statistical analyses

Analyses were conducted using the statistical program SPSS version 20.0 (SPSS Inc., 2011 (IBM SPSS, Armonk, New York, 2011)). All data were checked for normality and homogeneity of variance using Kolmogorov–Smirnov and Levene's tests. Data not meeting these assumptions were log‐transformed. Nonparametric tests were employed when transformation was not possible. SVL (33.18–42.40 mm; μ = 33.18 ± 2.49), ILL (17.34–24.55 mm; μ = 21.38 ± 1.99), and mass (0.78–1.85 g; μ = 1.31 ± 0.24), were not associated with any of the thermal traits (linear regression: *p *>* *.1), therefore these variables were not included in further analyses. Sprint speed values are presented in cm/s and *T*_set_'s are in number of degrees (°C). Statistical significance was assigned at α = 0.05 (Figure 1).

Pairwise correlations of thermal behavior traits were made using Spearman rank correlation. Separate principal component analyses (PCA) were performed for each time point to summarize traits into composite sets of variables. Estimation of relevant components to be extracted was based on the Kaiser–Guttman criterion (eigenvalues > 1). Individual scoring on the extracted components was estimated by the Anderson‐Rubin method. Contribution to each component >0.30 was considered significant (Tabachnick, Fidell, & Osterlind, 2001). Repeatability (τ) of PC scores (le Galliard et al., 2012) was calculated as the intraclass correlation coefficient using the variance components derived from one‐way ANOVAs (Lessells & Boag, 1987). Repeatable factors were used in determining the presence of a syndrome and describing its structure. Repeatability of individual traits was also calculated where sprint speed was assessed across test temperatures while temporal repeatability was assessed for all thermal traits.

## 3 Results

### 3.1 Thermal behavior syndrome

**Figure 2:**
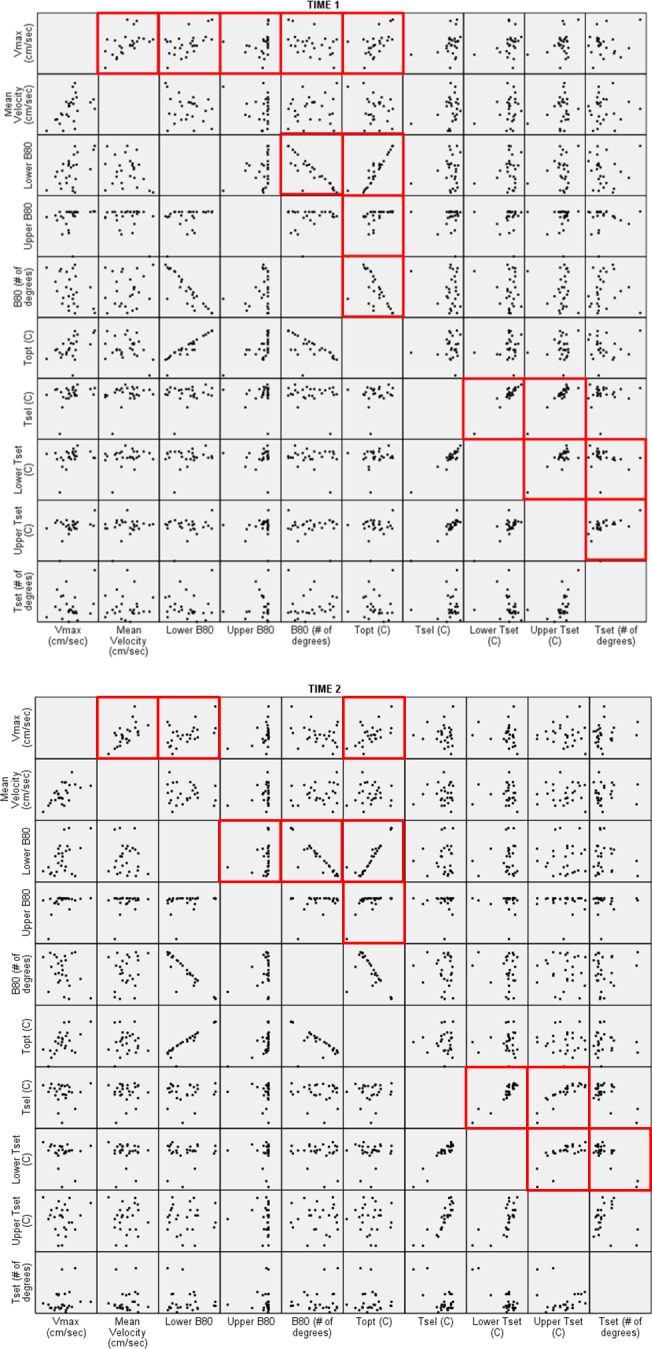
Scatterplot matrix of thermal behavioral traits at Time 1 (graph A) and Time 2 (graph B). Red boxes indicate significant correlations between traits

Within‐situation correlations varied between traits (Figure 2). More correlations occurred at Time 1 than Time 2. Maximum velocity was associated with the greatest number of traits during both time periods, including *V*_mean_ (Time 1: *r *=* *.565, *p *=* *.003; Time 2: *r *=* *.622, *p *=* *.001), LB_80_ (Time 1: *r *=* *.477, *p *=* *.014; Time 2: *r *=* *.426, *p *=* *.030), UB_80_ (Time 1: *r *=* *.542, *p *=* *.004), *B*_80_ (Time 1: *r *=* *−.399, *p *=* *.044), and *T*_opt_ (Time 1: *r *=* *.555, *p *=* *.003; Time 2: *r *=* *.498, *p *=* *.010). By contrast, *T*_set_ was only correlated with LT_set_ (*r *=* *−.417, *p *=* *.034) after the 9 weeks of acclimation. In general, lizards that ran fast had attained their maximal performance (*V*_max_) at high temperatures within a narrow range whereas slow lizards had relatively lower *T*_opt_'s that fell within a broad *B*_80_. Similarly, individuals that preferred higher *T*_b_'s had narrower setpoint ranges than those selecting lower *T*_b_'s.

Consistency in across‐context correlations among thermal traits suggests the presence of the TBS in that the PCA revealed a shared pattern of variation among thermal traits over 9 weeks of laboratory conditions. Four PCs were retained, however, only PC1 and PC3 were repeatable (τ = 0.04, *p *=* *.04; τ = 0.06, *p *=* *.01, respectively) (Table 2). Thus, the traits comprising each of these stable components served in describing the syndrome structure. PC1 explained the majority of the variation representing locomotor performance and thermal preferences whereas PC2 related mainly to performance breadth. Fast lizards that performed maximally at high temperatures within a narrow range also select high body temperatures which coincided with equally high lower and upper selected temperature ranges. Slow lizards, by contrast, had lower optimal and preferred body temperatures as well as broader ranges.

**Table 2:** Results of principal components analyses of locomotory performance and thermal preferenda measures at each time interval. Loadings, eigenvalues, and explained variance are given for repeatable PCs

Thermal behaviour trait	Time 1		Time 2	
	PC1	PC3	PC1	PC3
*V*_max_	0.57	−0.74		
*V*_mean_	0.64	−0.30		0.71
LB_80_	0.61		0.68	
UB_80_		0.76		0.64
*B*_80_	−0.63	0.50	−0.63	0.57
*T*_opt_	0.48		0.62	
*T*_sel_	0.82	0.36	0.75	
LT_set_	0.77	0.34	0.77	
UT_set_	0.78	0.42	0.56	
*T*_set_			−0.62	
Variance explained (%)	36.05	19.78	32.40	14.54

### 3.2 Repeatability of individual thermal traits

For the original thermal traits, sprint speed (*V*_mean_ and *V*_max_) was repeatable across the five test temperatures (τ = 0.06, *p *<* *.001; τ = 0.04, *p *=* *.01, respectively) with velocity being lowest at 15°C (σ = 24.52 ± 6.12 cm/s) and highest at 35°C (σ = 24.52 ± 6.12 cm/s) (Table 1). Similarly, *V*_mean_ and *V*_max_ also showed temporal stability between Time 1 and Time 2 (9 weeks) whereas all other performance measures (*T*_opt_, *B*_80_, LB_80_, and UB_80_) did not (Table 2). Among the four thermal preference measures, only *T*_sel_ and LT_set_ were repeatable (Table 2). Individual lizards consistently selected *T*_b_'s within the range of 17.16–28.14°C, preferences which fall within previous estimates for this species (18.6–33.6°C) (Greer, 1989). In contrast, the upper (UT_set_) set‐point temperature shifted from one time period to the next resulting in greater within‐individual variation in the setpoint range (*T*_set_).

## 4 Discussion

Adult delicate skinks exhibit consistent individual differences in thermal preference and locomotor performance traits. Moreover, stable across‐context correlations among these traits provided support for the proposed TBS whereby lizards ranked along a cold–hot continuum. Hot thermal types selected and performed maximally at higher *T*_b_'s whereas cold thermal types had lower *T*_sel_'s and *T*_opt_'s.

### 4.1 Interindividual variation in thermal traits

Both sprint speed (*V*_max_ and *V*_mean_) and thermal preferences (*T*_sel_ and LT_set_) were repeatable in this population of delicate skinks. Lizards that were fast at one temperature and time point were also fast later and at other temperatures while those that were slow remained slow. Similarly, lizards preferring a particular *T*_b_ maintained those preferences over the course of the study. Some individuals consistently selected temperatures which approached the upper bound (33.6°C) of this species’ normal range while others chose those that fell below the lower bound (18.6°C) suggesting the differentiation of lizards into particular thermal types. In other words, “hot” lizards stayed “hot” and “cold” lizards stayed “cold” even despite experiencing alterations in their thermal environments.

However, not all thermal traits were found to be repeatable and those that were varied in the degree of consistency. Divergent levels of stability among thermal traits could be attributable to the presence or absence of functional constraints (Adolph & Pickering, 2008; Careau & Garland, 2012). For example, daily variation in physiological state may limit the repeated expression of thermal preference traits. Higher levels of plasticity in upper thermal ranges, and thus set‐point range, would seemingly be advantageous as high body temperatures are required for such physiological processes as digestion, spermatogenesis, and behavioral fever (Angilletta, 2001; Seebacher & Franklin, 2005). Fluctuation in motivation levels may also cause trait expression to vary contextually, particularly with respect to locomotor performance. Here, habituation of the stimuli used to prompt running during the performance trials may have occurred, resulting in the perception of threat to be reduced. Conversely, *V*_mean_ as a less stringent measure reflecting the range of potential speeds in which a lizard can attain, thus, can be more readily consistent across time and context. Even in nature, lizards rarely perform at their maximum capacities or preferentially select precise *T*_b_'s, suggesting that these measures may not be directly related to fitness (Careau & Garland, 2012; Irschick, Herrel, Vanhooydonck, Huyghe, & van Damme, 2005). For example, maximum sprint speed does not predict survival for adult collared lizards (*Crotaphytus collaris*), but rather, survivorship is more reliant upon the proportion of their *V*_max_ used while escaping predators (Husak, 2006). This raises the possibility that natural selection may not target a single performance or *T*_b_, but instead, it may operate more strongly on some other broader metric such as realized sprint speed or thermal tolerance breadth (Husak, 2006).

The repeatable variation among individual thermal traits demonstrated here can be added to the mounting empirical evidence that selected body temperatures and locomotor performance among reptiles is independent of temporal and thermal factors. Our repeatability estimates for mean locomotor performance are similar to those reported in other studies of comparable experimental length. For instance, burst speed of the eastern garter snake (*Thamnophis sirtalis*) was repeatable (*r *=* *.34) over the course of 8 weeks (Jayne & Bennett, 1990). Similarly, western fence lizards (*Sceloporous occidentalis*) exhibited consistent sprint speed and stamina for 2 months (*r *=* *.37), and this individual consistency was maintained for over a full year (*r *=* *.18) (van Berkum et al., 1989). By contrast, thermal preference estimates were appreciably lower in the delicate skink than in other species of lizards (Artacho et al., 2013; le Galliard, le Bris, & Clobert, 2003; Stapley, 2006). This disparity, however, may simply be a reflection of the longer period between repeated measures in our study relative to previous studies. Increased time intervals have been shown to reduce repeatability (Bell, Hankison, & Laskowski, 2009). Nonetheless, it is striking that despite variation in *T*_b_, ontogenetic stage, body condition, reproductive state, and potentially energy reserves and motivation across these aforementioned species, individual consistency is still exhibited (Bennett, 1980; van Berkum et al., 1989).

### 4.2 Thermal behavior syndrome

Stability in across‐context correlations among the thermal traits revealed the presence of the TBS. Lizards were positioned along a cold–hot continuum varying in sprint performance and thermal preferences. As predicted, hot thermal types ran faster within a narrow range of temperatures and selected higher *T*_b_s, than did cold thermal types. Rank‐order positions along the cold–hot continuum remained consistent even after extensive time (9 weeks) under standard laboratory conditions. These results counter the key assumption of the coadaptation theory which states that changes in environmental temperatures should drive a corresponding shift in thermal traits at both the short‐term (acclimation) and long‐term (adaptation) levels as a means of optimizing physiological and locomotory function and ultimately fitness (Angilletta et al., 2006). In other words, it would be expected that hot thermal types should respond to the new, cooler environmental conditions with which they are experiencing by reducing the temperatures they select and perform maximally. Instead, the thermal behavioral traits of the delicate skink remained stable at this within‐generational level, irrespective of apparent changes in their thermal environments. Although the coadaptation hypothesis has some support (Bauwens et al., 1995; Kaufmann & Bennett, 1989), other studies are in alignment with our own results (van Berkum et al., 1989; Garland, 1991; Hertz, Huey, & Nevo, 1983) suggesting that the TBS may be preventing the independent expression of optimal thermal responses among other taxa as well.

From an ecological standpoint, the TBS could have significant effects on the behavior, community dynamics, and distribution of the delicate skink. Hot types, with their narrow performance temperatures, would likely be restricted to habitats with high thermal regimes and/or optimal thermoregulatory opportunities. Within such sites, hot lizards would likely spend a large proportion of time basking to attain their high *T*_opt_'s and surface activity would also likely be high as their fast sprint speed would reduce their vulnerability to predation thereby enabling them to spend the majority of their time acquiring key resource (e.g., prey, mates). Cold types, on the other hand, could potentially occupy a wider array of habitat types and invariably become more widespread given their broader range of *T*_sel_'s. However, lower thermal preferences and slower sprint speeds could force cold lizards to have reduced activity periods to avoid overheating or falling victim to predation. Alternatively, they could instead be restricted to predator‐free sites having greater access to refuge. The TBS may, therefore, promote lizards to engage in this intraspecific physiological niche‐partitioning by selecting environments which encourages the maintenance of their particular thermal type (Stamps & Groothuis, 2010).

So why then would the TBS even exist? And what mechanisms would serve to maintain this potentially suboptimal physiological strategy? Incubation temperature, and its underlying link with metabolic rate, may be the primary reason as to why thermal physiology would vary among individuals in a population (Biro & Stamps, 2010; Careau, Thomas, Humphries, & Réale, 2008). A positive feedback loop involving these two traits could very well establish the physiological trajectory of an individual, serving to stabilize its thermal traits across subsequent ontogenetic stages and ecological contexts (Sih & Bell, 2008; Sih, Bell, Johnson, & Ziemba, 2004). Under this developmental perspective, once the organizational effects of incubation temperature are established, constraints act in maintaining an individual's thermal type over time and context. Each trait is controlled by distinct components (e.g., pineal complex (Tosini, 1997); transient receptor potential ion channels (Seebacher, 2009) which together integrate as a whole to produce the thermoregulatory and metabolic systems driving thermal traits. A change in one component would then necessitate a concomitant changes in another, resulting in a system‐wide reorganization (Duckworth, 2010). Modification to any single thermal trait would thus be slow and incur substantial energetic costs. Having a fixed thermal type put forth from developmental pathways may, therefore, outweigh the potential benefits of developing physiological flexibility.

Divergent incubation temperatures would therefore generate a variety of thermal types within a given population, each associated with their own distinct activity level, food requirements, habitat use, and personality (Biro & Stamps, 2010; Careau & Garland, 2012; Pruitt et al., 2011; Reale et al., 2010). Accordingly, hot thermal types developing under high temperature conditions would presumably have high MRs supported by larger, more efficient metabolic machineries (Biro & Stamps, 2008; Reale et al., 2010). This greater aerobic capacity would promote a more active lifestyle, particularly with respect to those behaviors that maximize food intake, such as locomotion, exploration, aggression, and boldness (Biro & Stamps, 2010; Clarke & Fraser, 2004; Stapley, 2006). Thermal preferences would thus be equally high in order to assimilate adequate energy to support energetically expensive organs and activities (Careau & Garland, 2012). Conversely, cold thermal types experiencing low incubation temperatures would instead have low thermal traits, MRs, energy need, low activity, and small organ sizes. In having divergent food requirements, behavior, and habitat preferences, each thermal type would be best suited for different situations and environments, thereby maintaining their long‐term coexistence (Sih & Bell, 2008).

## 5 Conclusion

We have found compelling evidence for the presence of the TBS in the delicate skink. Within this syndrome, individuals vary in their thermal type as each is positioned along a cold–hot continuum based on their thermal preference and locomotory performance. As the first study of its kind, it provides a promising new framework for future investigations examining the correlation among physiological traits using a syndrome approach. These results could ultimately be combined with future investigations on the development of physiological variation as a way of determining the relative importance of constraints and selection in the evolution of thermal traits.

## Conflict of Interest

None declared.
