# Supplementary material for: Repeatability and correlation of physiological traits: Do ectotherms have a “thermal type”?
Source: Ecol Evol. 2016 Dec 22;7(2):710–9. doi: 10.1002/ece3.2632 (PMC5243194; doi:10.1002/ece3.2632)
Supplement: Supplementary file 1 [file ECE3-7-710-s001.docx]

**REPEATABILITY AND CORRELATION OF PHYSIOLOGICAL TRAITS: DO ECTOTHERMS HAVE A ‘THERMAL TYPE’?**

Celine T. Goulet, Michael B. Thompson, and David G. Chapple

**APPENDIX**

| **Table A1.** Summary of empirical studies presenting performance and thermal preferenda repeatabilities in ectothermic species. Repeatabilities varied between Pearson product-moment, Spearman rank-order correlation coefficients or Intra-class coefficients derived from one-way ANOVAS. Astricts denote significant results. | | | | | | |  |
| --- | --- | --- | --- | --- | --- | --- | --- |
|  | **Thermal Trait** | | ***N*** | **Repeatability** | **Time Period** | **Source** |  |
|  | ***Locomotory Performance*** | |  |  |  |  |  |
|  |  | eastern fence lizard (*Sceloporus undulatus*) | 14 | 0.50* | 1 week | Angilletta *et al.* 2002 |  |
|  |  | common lizard (*Zootoca vivipara*) | 12 | 0.38-0.52* | 2 days | Artacho *et al*. 2013 |  |
|  |  | California tiger salamander (*Ambystoma californiense*) | 90 | 0.22-0.67* | < 1 day | Austin and Shaffer 1992 |  |
|  |  | California tiger salamander (*A. californiense*) | 90 | 0.26-0.34 | 15 months | Austin and Shaffer 1992 |  |
|  |  | side-blotched lizard (*Uta stansburiana*) | 24 | 0.08-0.68* | 2 days | Brandt and Allen 2004 |  |
|  |  | northern curly-tailed lizard (*Leiocephalus carinatus*) | 45 | 0.71* | 1 - 11 days | Diamond *et al*. 2014 |  |
|  |  | central netted dragon (*Ctenophorus nuchalis*) | 73 | 0.77* | 1 day | Garland 1985 |  |
|  |  | central netted dragon (*C. nuchalis*) | 73 | 0.97* | 8 weeks | Garland 1985 |  |
|  |  | western fence lizard (*S. occidentalis*) | 42 | 0.839* | 2 days | Garland *et al.* 1990 |  |
|  |  | eastern garter snake (*Thamnophis sirtalis*) | 249 | 0.69-0.80* | 1 day | Garland Jr 1988 |  |
|  |  | canyon lizard (*S. merriami)* | 132 | 0.56-0.63* | 1 year | Huey and Dunham 1987 |  |
|  |  | roughtail rock agama (*Stellio stellio*) | 45 | 0.76-0.85 | 12 days | Huey and Hertz 1984 |  |
|  |  | *Agama savignyi* | 10 | 0.69 | 12 days | Huey and Hertz 1984 |  |
|  |  | canyon lizard (*S. merriami)* | 86 | 0.61 | 1 year | Huey and Bennett 1990 |  |
|  |  | eastern garter snake (*T. sirtalis*) | 274 | 0.62-0.78* | 1 day | Jayne and Bennett 1990 |  |
|  |  | eastern garter snake (*T. sirtalis*) | 86 | 0.34* | 8 weeks | Jayne and Bennett 1990 |  |
|  |  | eastern garter snake (*T. sirtalis*) | 67 | 0.25-0.65* | 1 year | Jayne and Bennett 1990 |  |
|  |  | eastern garter snake (*T. sirtalis*) | 41 | -0.12-0.18 | 2 years | Jayne and Bennett 1990 |  |
|  |  | eastern garter snake (*T. sirtalis*) | 11 | -0.52 | 3 years | Jayne and Bennett 1990 |  |
|  |  | western toad (*Bufo boreas*) | 10 | 0.51* | 1 day | Putnam and Bennett 1981 |  |
|  |  | western fence lizard (*S. occidentalis*) | 118 | 0.37* | 2 months | van Berkum *et al.* 1989 |  |
|  |  | western fence lizard (*S. occidentalis*) | 118 | 0.47* | 7 months | van Berkum *et al.* 1989 |  |
|  |  | western fence lizard (*S. occidentalis*) | 118 | 0.18* | 13 months | van Berkum *et al.* 1989 |  |
|  |  | Fowler's toad (*B. fowleri*) | 36 | 0.10-0.46* | 1 day | Walton 1988 |  |
|  | ***Preferred Body Temperature*** | |  |  |  |  |  |
|  |  | common lizard (*Z. vivipara*) | 22 | 0.60* | 1 month | Artacho *et al*. 2013 |  |
|  |  | cape girdled lizard (*Cordylus cordylus*) | 11 | 0.36* | within-day | Clusella Trullas *et al*. 2006 |  |
|  |  | black girdled lizard (*C. niger*) | 10 | 0.15* | within-day | Clusella Trullas *et al*. 2006 |  |
|  |  | Karoo girdled lizard (*C. polyzonus*) | 11 | 0.20* | within-day | Clusella Trullas *et al*. 2006 |  |
|  |  | Oelofsen's girdled lizard (*C. oelofseni*) | 10 | 0.21* | within-day | Clusella Trullas *et al*. 2006 |  |
|  |  | cape girdled lizard (*Cordylus cordylus*) | 11 | 0.26* | 1 week | Clusella Trullas *et al*. 2006 |  |
|  |  | black girdled lizard (*C. niger*) | 10 | 0.00 | 1 week | Clusella Trullas *et al*. 2006 |  |
|  |  | Karoo girdled lizard (*C. polyzonus*) | 11 | 0.08 | 1 week | Clusella Trullas *et al*. 2006 |  |
|  |  | Oelofsen's girdled lizard (*C. oelofseni*) | 10 | 0.06 | 1 week | Clusella Trullas *et al*. 2006 |  |
|  |  | marine toad (*B. marinus*) | 34 | 0.66* | 5 days | Dohm *et al.* 2001 |  |
|  |  | *common lizard* (*Lacerta vivipara*) | 19 | 0.66 | 2 days | Le Galliard *et al*. 2003 |  |
|  |  | *mountain log skink (Pseudomoia entrecasteauxii)* | 32 | 0.48-0.58 | 3 days | Stapley 2006 |  |
|  |  | armadillo girdled lizard (*Ouroborus cataphractus*) coastal population | 5 | 0.24* | 13 days (autumn) | Truter *et al*. 2014 |  |
|  |  | armadillo girdled lizard (*O. cataphractus*) coastal population | 7 | 0.33* | 13 days (spring) | Truter *et al*. 2014 |  |
|  |  | armadillo girdled lizard (*O. cataphractus*) inland population | 6 | 0.44* | 13 days (autumn) | Truter *et al*. 2014 |  |
|  |  | armadillo girdled lizard (*O. cataphractus*) inland population | 6 | 0.63* | 13 days (spring) | Truter *et al.* 2014 |  |
|  |  | |  |  |  |  |  |

**References**

ARTACHO, P., JOUANNEAU, I. & LE GALLIARD, J. F. 2013. Interindividual variation in thermal sensitivity of maximal sprint speed, thermal behavior, and resting metabolic rate in a lizard. *Physiol Biochem Zool,* 86**,** 458-69.

AUSTIN, C. & SHAFFER, H. 1992. Short-, medium-, and long-term repeatability of locomotor performance in the tiger salamander Ambystoma californiense. *Functional Ecology,* 6**,** 145-153.

BRANDT, Y. & ALLEN, J. R. 2004. Persistence of individually distinctive display patterns in fatigued side-blotched lizards (*Uta stansburiana*). *Behavioral Ecology and Sociobiology,* 55**,** 257-265.

CLUSELLA TRULLAS, S., TERBLANCHE, J. S., WYK, J. H. & SPOTILA, J. R. 2006. Low repeatability of preferred body temperature in four species of Cordylid lizards: Temporal variation and implications for adaptive significance. *Evolutionary Ecology,* 21**,** 63-79.

DIAMOND, K., TROVILLION, D., ALLEN, K. E., MALELA, K. M., NOBLE, D. A., POWELL, R., EIFLER, D. A. & GIFFORD, M. E. 2014. Individual (co)variation of field behavior and locomotor performance in curly tailed lizards. *Journal of Zoology,* 294**,** 248-254.

DOHM, M. R., MAUTZ, W. J., LOOBY, P. G., GELLERT, K. S. & ANDRADE, J. A. 2001. Effects of Ozone on Evaporative Water Loss and Thermoregulatory Behavior of Marine Toads (Bufo marinus). *Environmental research,* 86**,** 274-286.

GARLAND JR, T. 1988. Genetic basis of activity metabolism. I. Inheritance of speed, stamina, and antipredator displays in the garter snake *Thamnophis sirtalis*. *Evolution,* 42**,** 335-350.

GARLAND JR, T., HANKINS, E. & HUEY, R. 1990. Locomotor capacity and social dominance in male lizards. *Functional Ecology,* 4**,** 243-250.

GARLAND, T. 1985. Ontogenetic and individual variation in size, shape and speed in the Australian agamid lizard Amphibolurus nuchalis. *Journal of Zoology,* 207**,** 425-439.

HUEY, R. B. & DUNHAM, A. E. 1987. Repeatability of locomotor performance in natural populations of the lizard *Sceloporus merriami*. *Evolution,* 41**,** 1116-1120.

HUEY, R. B. & HERTZ, P. E. 1984. Is a jack-of-all-temperatures a master of none? *Evolution,* 38**,** 441-444.

JAYNE, B. C. & BENNETT, A. 1990. Scaling of speed and endurance in garter snakes: a comparison of cross‐sectional and longitudinal allometries. *Journal of Zoology,* 220**,** 257-277.

LE GALLIARD, J. F., LE BRIS, M. & CLOBERT, J. 2003. Timing of locomotor impairment and shift in thermal preferences during gravidity in a viviparous lizard. *Functional Ecology,* 17**,** 877-885.

PUTNAM, R. W. & BENNETT, A. F. 1981. Thermal dependence of behavioural performance of anuran amphibians. *Animal Behaviour,* 29**,** 502-509.

STAPLEY, J. 2006. Individual variation in preferred body temperature covaries with social behaviours and colour in male lizards. *Journal of Thermal Biology,* 31**,** 362-369.

TRUTER, J. C., VAN WYK, J. H. & MOUTON, P. L. F. N. 2014. An evaluation of daily, seasonal and population-level variation in the thermal preference of a group-living lizard, Ouroborus cataphractus (Sauria: Cordylidae). *Amphibia-Reptilia,* 35**,** 391-403.

VAN BERKUM, F., HUEY, R., TSUJI, J. & GARLAND, T. 1989. Repeatability of individual differences in locomotor performance and body size during early ontogeny of the lizard *Sceloporus occidentalis* (Baird & Girard). *Functional Ecology,* 3**,** 97-105.

WALTON, M. 1988. Relationships among metabolic, locomotory, and field measures of organismal performance in the Fowler's toad (*Bufo woodhousei fowleri*). *Physiological Zoology,* 61**,** 107-118.
